# Shielding evaluation for IMRT implementation in an existing accelerator vault

**DOI:** 10.1120/jacmp.v4i3.2520

**Published:** 2003-06-01

**Authors:** R. A. Price, O. Chibani, C.‐M. Ma

**Affiliations:** ^1^ Department of Radiation Oncology Fox Chase Cancer Center 7701 Burholme Avenue Philadelphia Pennsylvania 19111

**Keywords:** IMRT, shielding, neutrons, Monte Carlo

## Abstract

A formalism is developed for evaluating the shielding in an existing vault to be used for IMRT. Existing exposure rate measurements are utilized as well as a newly developed effective modulation scaling factor. Examples are given for vaults housing 6, 10 and 18 MV linear accelerators. The use of an 18 MV Siemens linear accelerator is evaluated for IMRT delivery with respect to neutron production and the effects on individual patients. A modified modulation scaling factor is developed and the risk of the incurrence of fatal secondary malignancies is estimated. The difference in neutron production between 18 MV Varian and Siemens accelerators is estimated using Monte Carlo results. The neutron production from the Siemens accelerator is found to be approximately 4 times less than that of the Varian accelerator resulting in a risk of fatal secondary malignancy occurrence of approximately 1.6% when using the SMLC delivery technique and our measured modulation scaling factors. This compares with a previously published value of 1.6% for routine 3D CRT delivery on the Varian accelerator.

PACS number(s): 87.52.Ga, 87.52.Px, 87.53.Qc, 87.53.Wz

## INTRODUCTION

It is evident that while the use of intensity modulated radiation therapy (IMRT) yields desirable dose distributions, the delivery is inefficient. Followill *et al.* ^1^ estimated that the required number of monitor units (MU) for an IMRT treatment increase by a factor of 2 to 5 over conventional techniques. This increase in MU has a direct impact on the radiation shielding of the vault housing the linear accelerator used to deliver IMRT. Mutic *et al.* ^2^ and Rodgers^3^ have described methods to calculate the required shielding when designing a vault to be used for IMRT. We believe that the vast majority of centers implementing IMRT will do so in existing vaults rather than design and build new vaults specifically for this purpose. With this in mind we have developed a method to allow the shielding in an existing vault to be evaluated for IMRT delivery. This method incorporates previously measured values of exposure rate and comparisons are made with values used in the shielding design of the associated vault.

Subsequent to developing this method for shielding evaluation, we have applied it to the evaluation of an 18 MV Siemens Primus linear accelerator used for delivering IMRT. Neutron production in this accelerator is evaluated using data from the National Council on Radiation Protection and Measurements (NCRP) Report No. 79^4^ as well as full Monte Carlo head simulations. The effects on vault shielding as well as the increased probability of induction of secondary malignancies are discussed.

## METHODS

**Figure 1:**
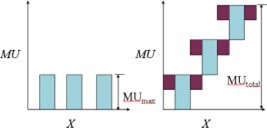
(Color) The diagram on the left depicts the desired intensity pattern and associated MU. The individual spaces between the bars correspond to areas that would be “blocked” in a conventional treatment. The diagram on the right depicts the same intensity pattern to be delivered using a MLC.

IMRT is delivered at this center using SMLC (segmental multi‐leaf collimation).^5^ With this delivery method the beam is “off” (no radiation being produced) while the MLC and/or gantry is in motion. The individual leaves move into or out of the treatment field along a single direction with respect to each segment. In order to create the complex intensity maps required, the MLC must use a series of “stacked” segments, or individual fields atop one another. Each of these individual segments is assigned its own monitor unit (MU) setting corresponding to the intensity map and associated with the dose required. Since the MLC moves in a single direction it is impossible to create “island blocks” and necessitates using multiple segments resulting in an increased number of MU versus a standard or transmission block. The ratio between the maximum number of MU required by the intensity map and the total MU needed to achieve the subsequent patterns is known as the modulation scaling factor (MSF).^6^ Figure 1 illustrates the MSF. In this illustration an IMRT treatment would require three times more MU than the conventional treatment yielding a MSF of 3.

(1) $$\text{MSF}={\text{MU}}_{\text{total}}/{\text{MU}}_{\text{max}}=3/1=3.$$

**Figure 2:**
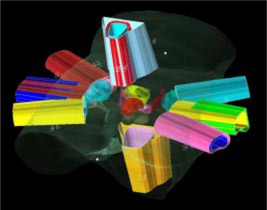
(Color) Illustration of previous 3D CRT technique used at FCCC Treatment included initial four‐field box to small pelvis, four‐field box cone‐down to prostate and seminal vessicals, five‐field non‐coplanar cone‐down to prostate only, and five‐field non‐coplanar cone‐down to prostate only with reduced margins.

For practical purposes we have modified this equation as:

(2) $${\text{MSF}}_{\text{mod}}={\text{MU}}_{\text{IMRT}}/{\text{MU}}_{\text{3D}\;\text{CRT}},$$

where ${\text{MU}}_{\text{IMRT}}$ is the total number of MU for a daily IMRT treatment and ${\text{MU}}_{3D\;\text{CRT}}$ is the total number of MU for a daily treatment planned using conventional 3D methods. We have calculated the values of the ${\text{MSF}}_{\text{mod}}$ for treatments delivered on the Primus units at this institution for 6, 10, and 18 MV. The calculations are based on prostate treatments since over 90% of the approximate 900 patients treated with IMRT at this institution were treated for this disease. All IMRT plans were generated using the Corvus treatment planning system (NOMOS Corp., Sewickley, PA). A series of ten patients at each of the three photon energies who had received IMRT treatment were randomly chosen and their resultant total MU tallied. Additionally, each of these patients was evaluated using our previous 3D CRT (three‐dimensional, conformal radiation therapy) technique which included treatment to the small pelvis via a four‐field “box” technique to 56 Gy followed by a complex five‐field non‐coplanar technique for an additional 22 Gy. All 3D CRT treatments were normalized to the 95% isodose line. Figure 2 illustrates this technique.

Subsequently, we have further modified the MSF to account for IMRT delivery efficiency. The average time for delivery was derived for the ten patients evaluated at each energy. This time includes MU delivery, MLC configuration, and gantry rotation^7^ Additionally, we localize the prostate prior to each treatment for each patient using the B‐mode acquisition and targeting (BAT) ultrasound device (NOMOS corp., Sewickley, PA). The resulting average localization time is added to the setup and delivery time to derive an average overall treatment time per patient. The maximum number of patients per hour (# patients/hr) that can be treated with IMRT is calculated. We then define the effective MSF (${\text{MSF}}_{\text{effective}}$) as

(3) $${\text{MSF}}_{\text{effective}}=\frac{\left(\text{Ave}\;\text{MU}/\text{pt}\right)\left(\#\;\text{patients}/\text{hr}\right)\left(40\;\text{hrs}/\text{wk}\right)}{W}.$$

The number of patients treated per hour is found to be dose rate dependant. The Primus linear accelerator delivers radiation at a rate of 300 MU/min for 6 MV and 500 MU/min for 10 and 18 MV in our current configuration. A typical work week for a radiation therapist is 40 hours. The dose per week that the vault was originally shielded for or workload (W) is 50,000 cGy^8^ at this institution and as a first approximation for shielding purposes 1 cGy is considered to be equivalent to 1 MU.

We can now evaluate the vault shielding under IMRT conditions by applying our ${\text{MSF}}_{\text{effective}}$ to our measured exposure rate readings. Equation (4) illustrates how the ${\text{MSF}}_{\text{effective}}$ is used to scale the weekly exposure rate previously determined for conventional delivery methods during accelerator installation to derive the weekly exposure rate for IMRT

(4) $${\left(\text{Weekly}\;\text{exposure}\;\text{rate}\right)}_{\text{IMRT}}=\left({\text{MSF}}_{\text{effective}}\right)\left(\text{Weekly}\;\text{exposure}\;\text{rate}\right).$$

The weekly exposure rates are derived from direct measurement at points surrounding the accelerator vault. These exposure rates are typically measured for a series of gantry angles under extremely conservative conditions including a $40\times 40\;{\text{cm}}^{2}$ field size and a 45° collimator rotation. Measurements at points corresponding to secondary barriers also employ the use of a scattering phantom while points corresponding to primary barriers do not. Table I includes the measured exposure rates at the associated gantry angles for points corresponding to occupational locations surrounding one of our vaults housing a 6 MV Siemens accelerator. Additionally, the use factor (U) derived for each gantry angle is included. For brevity only eight points are listed. Note that the room is shielded following the “As Low As Reasonably Achievable” (ALARA) concept to keep the total exposure rate below 10 mR/wk (0.1 mSv/wk) at any one point. This is approximately ten times less than the maximum permissible dose (MPD). The total exposure rate is determined by the sum of the measured exposure rates at each point multiplied by that point's respective use factor.

(5) $$\text{Total}\;\text{exposure}\;\text{rate}=\Sigma \left[{\left(\text{exposure}\;\text{rate}\right)}_{\text{gantry}}(U_{\text{gantry}})\right].$$

**Table I:** Exposure rates (in mR/hr) surrounding vault housing a 6 MV Siemens linear accelerator at FCCC.

Gantry	*U*	Point	1	2	3	4	5	6	7	8
0	0.46		0.20	0.70	0.14	0.80	0.80	0.40	0.20	0.10
270	0.125		0.12	2.30	0.16	1.90	1.60	4.10	0.45	0.10
180	0.29		0.18	4.50	0.10	0.10	0.10	0.30	0.17	0.12
90	0.125		0.83	0.58	0.72	0.61	0.49	0.20	0.01	0.14
Total exposure rate			0.26	1.99	0.20	0.71	0.66	0.81	0.20	0.11

The weekly exposure rate is given by equation 6 where the workload is assumed to be 500 Gy/wk (50,000 MU/wk) and the dose rate is assumed to be 3 Gy/min (300 MU/min).

(6) Weekly exposure rate=(total exposure rate)(W)/[(Dose rate)(60 min/hr)].

Tables II and III include the measured exposure rates at the associated gantry angles for points corresponding to occupational locations surrounding vaults housing 10 and 18 MV Siemens accelerators respectively. The use factor derived for each gantry angle is included. For brevity only eight points are listed.

**Table II:** Exposure rates (in mR/hr) surrounding vault housing a 10 MV linear accelerator at FCCC.

Gantry	*U*	Point	1	2	3	4	5	6	7	8
0	0.337		0.14	0.22	0.31	0.09	0.09	0.09	0.15	0.18
270	0.198		0.12	0.30	0.75	0.13	0.07	0.15	0.85	0.30
180	0.267		0.12	0.20	0.33	0.07	0.09	0.10	0.40	0.26
90	0.198		0.40	3.20	11.70	1.90	8.50	11.50	29.00	1.50
Total exposure rate			0.18	0.82	2.66	0.45	1.75	2.36	6.07	0.49

**Table III:** Exposure rates (in mR/hr) surrounding vault housing an 18 MV linear accelerator at FCCC.

Gantry	*U*	Point	1	2	3	4	5	6	7	8
0	0.233		0.12	0.13	0.08	0.13	0.13	0.20	0.20	0.20
270	0.244		0.16	0.25	3.90	0.90	1.60	1.30	0.27	0.24
180	0.223		0.10	0.15	0.07	0.15	0.16	0.12	0.22	0.17
90	0.245		0.09	0.13	0.06	0.14	0.16	0.11	0.20	0.20
Total exposure rate			0.11	0.16	1.00	0.32	0.50	0.42	0.21	0.19

In order to assess the effect of IMRT delivery on neutrons produced through photo‐nuclear interactions at 18 MV, the ${\text{MSF}}_{\text{effective}}$ was applied to the measured neutron dose rate at points surrounding this vault. Points at the control console and at the vault door are evaluated.

## RESULTS

The average number of MU per patient under 3D CRT conditions for the 6 MV accelerator evaluated was 366 MU with a standard deviation (s.d.) of 12 MU (range 355–390 MU). For IMRT delivery the average increased to 1323 MU with a s.d. of 265 MU (range 884–1654 MU). The resultant ${\text{MSF}}_{\text{mod}}$ was determined to be 3.61 with a s.d. of 0.73 and a standard error (s.e.) of 0.23.

However, due to the dose rate of this machine (300 MU/min) we are limited to the number of patients we can treat per hour. The mean treatment time for the 10 IMRT patients evaluated was 16.5 minutes with a standard deviation of 5.3 minutes (range 7.7–24 min). Including time for target localization we can conservatively estimate the time for each treatment to be approximately 20 minutes or approximately 3 patients per hour. Subsequent calculations for ${\text{MSF}}_{\text{effective}}$ yield a value of 3.18. Applying the ${\text{MSF}}_{\text{effective}}$ to our weekly exposure rates as in Eq. (4) above yields weekly exposure rates under IMRT conditions. These data are given in Table IV.

**Table IV:** Weekly exposure rates (in mR/wk) under IMRT conditions for points surrounding vaults housing 6, 10, and 18 MV accelerators at FCCC. See text for explanation.

Point	1	2	3	4	5	6	7	8
6 MV	2.32	17.55	1.80	6.28	5.81	7.14	1.76	0.98
remeasured under IMRT conditions		3.30						
10 MV	1.14	5.11	16.57	0.18	0.68	0.92	2.36	0.19
remeasured under IMRT conditions		5.61						
18 MV	0.63	0.89	5.67	1.80	2.81	2.37	1.19	1.09

The design limit for each point was 10 mR/wk (0.1 mSv/wk). Point 2 corresponds to a treatment planning area adjacent to this vault and is assumed to have 100% occupancy. While the exposure rate under IMRT conditions is still approximately only 17.5% of the permissible limit, our design criteria have been exceeded. However, our original exposure rates were measured under extremely conservative conditions as mentioned previously. It is unlikely to have individual segments as large as $40\times 40\;{\text{cm}}^{2}$ for IMRT. Subsequently, the exposure rates were re‐measured at this point using a more realistic $10\times 10\;{\text{cm}}^{2}$ field size. The resultant weekly exposure rate was determined to be 1.04 mR/wk (10.4 μSv/wk) and after applying our ${\text{MSF}}_{\text{effective}}$ the exposure rate at this point is approximately 3.30 mR/wk (33 μSv/wk) under IMRT conditions.

For the 10 MV beam, the average number of MU per patient under 3D CRT conditions was 295 MU with a s.d. of 12 MU (range 278–321 MU). For IMRT delivery the average increased to 1168 MU with a s.d. of 139 MU (range 972–1367 MU). The resultant ${\text{MSF}}_{\text{mod}}$ was determined to be 3.95 with a s.d. of 0.40 and a s.e. of 0.13. Due to the dose rate of this machine (500 MU/min) we are still limited to the number of patients we can treat per hour. The mean treatment time for the 10 IMRT patients evaluated was 10.4 min with a s.d. of 3.1 min (range 7.3–17.2 min). Including time for localization we can conservatively estimate the time for each treatment to be approximately 15 min or approximately four patients per h. Subsequent calculations for ${\text{MSF}}_{\text{effective}}$ yield a value of 3.74. Applying the ${\text{MSF}}_{\text{effective}}$ to our weekly exposure rates as in Eq. (4) above yields weekly exposure rates under IMRT conditions. These data are given in Table IV.

Point 3 corresponds to a corner in the accelerator control area and is assumed to have 100% occupancy. While the exposure rate under IMRT conditions is still approximately only 16.6% of the permissible limit, our design criteria have been exceeded. We remeasured using the more realistic $10\times 10\;{\text{cm}}^{2}$ field size. The resultant weekly exposure rate was determined to be 1.50 mR/wk (15 μSv/wk) and after applying our ${\text{MSF}}_{\text{effective}}$ the exposure rate at this point is approximately 5.61 mR/wk (56.1 μSv/wk) under IMRT conditions.

For the 18 MV beam, the average number of MU per patient under 3D CRT conditions was 274 MU with a s.d. of 11 MU (range 256–297 MU). For IMRT delivery the average increased to 1063 MU with a s.d. of 111 MU (range 866–1225 MU). The resultant ${\text{MSF}}_{\text{mod}}$ was determined to be 3.89 with a s.d. of 0.43 and a s.e. of 0.14. The dose rate of this machine is 500 MU/min. The mean treatment time for the 10 IMRT patients evaluated was 11.7 min with a s.d. of 2.5 min (range 7.8–15.2 min). Including time for localization we can conservatively estimate the time for each treatment to be approximately 15 min or approximately four patients per hour. Subsequent calculations for ${\text{MSF}}_{\text{effective}}$ yield a value of 3.40. Applying the ${\text{MSF}}_{\text{effective}}$ to our weekly exposure rates as in Eq. (4) above yields weekly exposure rates under IMRT conditions. These data are given in Table IV.

The neutron dose rate at the console area was measured as 0.8 μrem/500 MU. Neutron measurements were obtained using a Neutron/Gamma Survey Meter model NG‐2A (Nuclear Research Corporation). Application of our method is demonstrated in Eq. (7) where 500 Gy is the dose per week that the room is shielded for and *Q* is the quality factor for fast neutrons and is given a value of 20 (Ref. 9) and 0.054 mSv/wk is the weekly neutron dose rate under IMRT conditions.

(7) $$(\text{Neutron}\;\text{dose}\;\text{rate})({\text{MSF}}_{\text{effective}})\mathrm{WQ}=0.054\;\text{mSv}/\text{wk}.$$

The measured neutron dose rate at the vault door (closed) was found to be 4.75 μrem/500 MU. Application of Eq. (7) yields a weekly neutron dose rate of 0.323 mSv/wk.

## DISCUSSION

The evaluation of the shielding in an existing accelerator vault under IMRT conditions will undoubtedly present numerous questions. One such question would be what to do if the adjusted exposure rates are less than those allowed by the MPD but exceed the values the room was originally shielded for? Obviously, shielding could be added in the required areas although this could be quite expensive. An alternative approach may be to limit the number of IMRT patients treated per day on the accelerator in question until the combination of conventional and IMRT treatments yield acceptable exposure rate limits. As seen in our 6 and 10 MV examples above, we have chosen to re‐measure the exposure rates at the areas in question under conditions more indicative of IMRT delivery. If this had not produced the desired effect, to operate in compliance with State regulations we would have had to consider one of the other options. However, we would caution the reader to contact his/her regulator on a case‐by‐case basis to determine the appropriateness of this method.

One general recommendation we can make in reference to shielding is to make efforts to reduce the ${\text{MSF}}_{\text{effective}}$ whether through judicious beam direction selection, delivery technique, appropriate number of intensity levels used for segmentation, techniques for MU reduction, etc. Any or all of these methods will have a positive effect on the resultant exposure rates.

It has been suggested that 18 MV photons not be used for IMRT. Indeed, in reference to the occurrence of secondary fatal malignancies, Followill *et al.* state that “It is unlikely that either an 18‐ or 25‐MV x‐ray beam technique would be so extraordinarily superior to a conventional technique as to justify the increased risk.”^1^ They estimated that the risk of occurrence of secondary fatal malignancies would increase from 1.6% to 4.5% when comparing 18 MV conventional to MLC‐based IMRT treatments on Varian linear accelerators. A ${\text{MSF}}_{\text{mod}}$ of 2.8 was assumed in the above assessment. The increase in dose rate from 300 MU/min at 6 MV to 500 MU/min at 18 MV on an existing Siemens Primus linac in our department, prompted us to give consideration to the energy increase.

**Figure 3:**
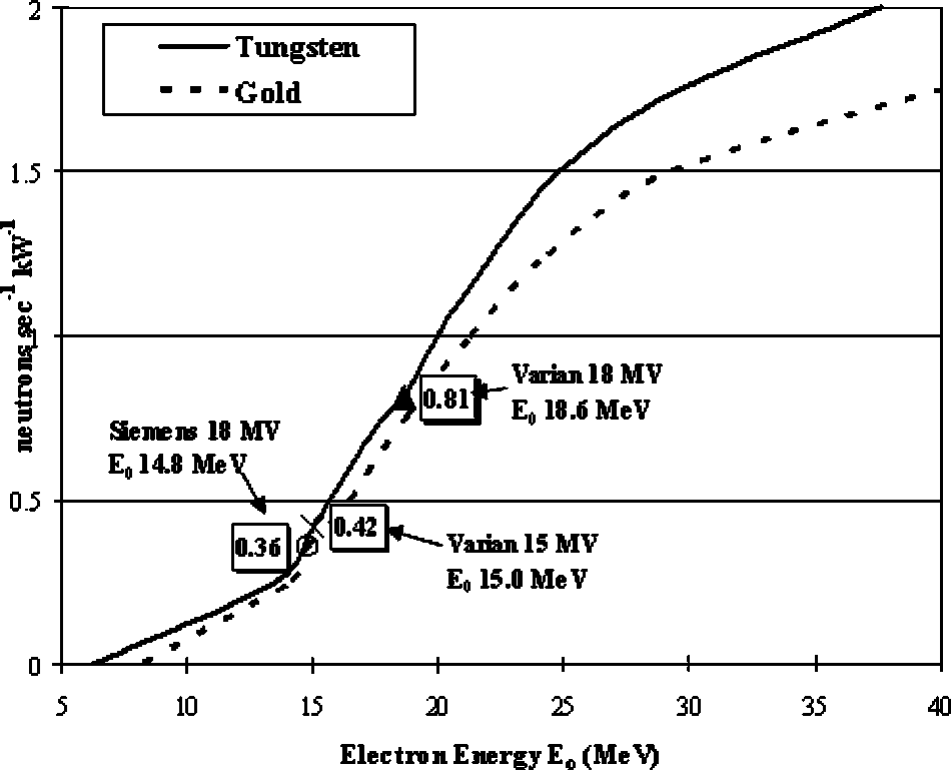
Neutron yields on semi‐infinite targets as a function of electron energy $E_{0}$ at the exit window in the accelerator head for both Siemens and Varian linacs operating at the listed nominal energies.

During the linac head modeling procedure for an unrelated Monte Carlo simulation project we became aware of a significant input electron energy difference between the nominal 18 MV Varian and Siemens linear accelerators. The input energy (18.6 MeV) for the Varian unit was higher than the nominal photon energy while lower (14.8 MeV) than the nominal photon energy for the Siemens unit. Neutron production is directly related to the input electron energy. Additionally, the majority of neutrons are produced in the target, flattening filter, collimators and shielding materials.^10^ As a first approximation we assumed single targets to be semi‐infinite and composed of a single material for each accelerator. The data in Fig. 3 were reproduced from NCRP report no. 79.^4^ It would appear that neutron production from a nominal 18 MV Siemens accelerator is approximately 2.25 times lower than the Varian accelerator with the same nominal photon energy. In fact neutron production is approximately equal to that of a nominal 15 MV Varian x‐ray beam.

We have estimated the risk of occurrence of secondary fatal malignancies resulting from 18 MV IMRT treatments on a Siemens linear accelerator following the same formalism as Followill *et al.* They give a neutron dose equivalent per photon cGy, 50 cm from the central axis in the patient plane of $4.6\times 10^{-2}$ mSv.^1^ No value is given for the Siemens linear accelerator at this nominal energy and we have simply applied our first approximation of the ratio between neutron production at 18 MV (0.444 Siemens/Varian). NCRP Report No. 116 gives a lifetime risk value of $5.0\times 10^{-2}$ ${\text{Sv}}^{-1}$ for fatal radiation induced cancers for the general population.^9^ Applying our derived ${\text{MSF}}_{\text{mod}}$ of 3.89 for 18 MV and 7600 cGy for a prostate treatment at our institution yields a 3.0% risk of induced fatal secondary malignancies. This would be an approximate two‐fold increase over conventional techniques delivered on a Varian accelerator versus the approximate three‐fold increase found for IMRT delivery on the Varian accelerators at a lower dose of 7000 cGy

To investigate further we performed Monte Carlo simulations of the above accelerators using the MCNPX code. The linac heads were simulated in detail.11 From this full head simulation it would appear that neutron production from a nominal 18 MV Siemens accelerator is approximately 4.06 times lower than the Varian accelerator with the same nominal photon energy. Following the same procedure as above and applying our derived ${\text{MSF}}_{\text{mod}}$ and 7600 cGy we find a risk of induced fatal secondary malignancy of 1.67%. This is comparable with the 1.6% increase determined by Followill *et al.* for conventional 3D CRT on a Varian 18 MV machine. In fact, it would appear from our simulations that the 18 MV Siemens beam generates fewer neutrons than the nominal 15 MV Varian beam by an approximate factor of 2.3.

This study was performed as a relative comparison between accelerators of two different manufacturers. The values used for lifetime risks for fatal radiation induced cancers for the general population and the neutron dose equivalent per photon cGy at isocenter contain inherent uncertainties. Additionally, due to the lower neutron production of the Siemens linear accelerator we could expect the rate of occurrence of secondary malignancies for conventional treatments to be lower than the 1.6% estimated for the Varian linear accelerator. The absolute value for difference in secondary malignancy occurrence between conventional techniques and IMRT would likely be related to the derived ${\text{MSF}}_{\text{mod}}$ for the Siemens units. As a safety precaution we have instituted an age limit of ≥65 years of age for patients to be treated with IMRT using Siemens 18 MV photons.

## CONCLUSIONS

The majority of the shielding design calculations for existing accelerator vaults probably did not take into account the effects of IMRT. Each accelerator/vault combination should be evaluated prior to implementation of this delivery technique. We have described a method for this evaluation that is straightforward and utilizes previously measured weekly exposure rate values. Additionally, we have suggested methods to follow should the weekly exposure rate values under IMRT conditions exceed the values the room was initially shield for.

As with vault shielding under IMRT conditions, the choice of nominal photon energy should be properly evaluated prior to utilization. We have demonstrated that while the use of 18 MV photons for IMRT may result in an increase in the induction of fatal secondary malignancies from neutron exposure over conventional delivery techniques, the absolute value of induction is manufacturer dependant. The implementation of IMRT presents a host of issues including shielding and the use of high energy beams that should be addressed in each individual clinic.
